# Epidemiological and clinical characteristics of pediatric corrosive ingestion and factors associated with acute esophageal injury: a single-center retrospective study

**DOI:** 10.3389/fphys.2026.1807937

**Published:** 2026-05-29

**Authors:** Yingru Liu, Siyue Tang, Hao He, Hong Mei, Baoxiang Wang, Dan Luo

**Affiliations:** 1 Medical Department of School of Medicine, Wuhan University of Science and Technology, Wuhan, China; 2 Joint Training Base for Postgraduate Students, Wuhan University of Science and Technology & Wuhan Children's Hospital, Wuhan, China; 3 Department of Gastroenterology, Wuhan Children’s Hospital, Tongji Medical College, Huazhong University of Science and Technology, Wuhan, China

**Keywords:** burns, chemical, caustics, children, epidemiology, esophageal stenosis, risk factors

## Abstract

**Background:**

This study aimed to investigate the epidemiology and clinical characteristics of accidental corrosive agent ingestion in children and to identify factors associated with the severity of acute corrosive esophageal injury.

**Methods:**

We retrospectively analyzed demographic and clinical data of 138 children who accidentally ingested corrosive agents in a single center from July 2014 to October 2024. The severity of esophageal injury was assessed using the Zargar grading system (Grade 0-IV) during initial endoscopy. Univariate analysis and multivariate logistic regression were used to identify factors associated with acute esophageal injury.

**Results:**

The median age of the 138 children was 2.08 years (interquartile range: 1.54–3.27). Children aged ≤5 years accounted for 87.68% (121/138), with a male-to-female ratio of 2:1. Alkaline agents accounted for 75.36% (104/138), with pipe cleaners being the most common source (28.99%). Gastrointestinal symptoms were the most frequent presentation (42.03%), followed by oral symptoms (29.71%), while 28.26% were asymptomatic. The median hospital stay was 2 days. Initial endoscopy showed normal mucosa (Grade 0) in 98 cases (71.01%) and abnormal findings (Grade I–IIIb) in 40 cases (28.99%). Proton pump inhibitors were used in 127 cases (92.03%), and corticosteroids in 48 cases (34.78%). Univariate analysis revealed significant correlations between abnormal Zargar grade and both age and time from ingestion to hospital presentation (both *P* < 0.05). Multivariate logistic regression further identified that age ≤5 years (odds ratio = 0.30, 95% confidence interval: 0.10–0.86, *P* = 0.025) and time to presentation ≤6 hours (odds ratio = 0.43, 95% confidence interval: 0.19–0.96, *P* = 0.038) were independent protective factors against abnormal endoscopic findings. Among the 17 patients who completed follow-up (12.32%), 9 (52.94%) developed dysphagia and 8 (47.06%) developed esophageal strictures; however, the low follow-up rate and selection bias limit the generalizability of these findings.

**Conclusion:**

Age >5 years and time from ingestion to hospital presentation >6 hours are associated with an increased risk of acute esophageal injury in children following accidental corrosive ingestion (odds ratio = 3.33 and 2.33, respectively). Prompt medical evaluation and endoscopic assessment are recommended for children with suspected ingestion.

## 1 Introduction

Ingestion of corrosive substances is a common accidental injury in children, which can lead to corrosive esophageal injury (CEI). Epidemiological data show an incidence ranging from 5 to 518 per 100,000 individuals annually (Arnold and Numanoglu, 2017). The severity of esophageal damage is determined by the physicochemical properties of the corrosive agent and the duration of exposure (Irlayıcı et al., 2025). In the early stage, the injury is characterized mainly by congestion, edema, erosion, ulceration, necrosis, and even perforation of the esophageal mucosa, whereas esophageal stricture may develop in the late stage (Eke et al., 2023). Currently, upper gastrointestinal endoscopy serves as an important tool for assessing the extent and severity of esophageal injury (Datta et al., 2024; Li et al., 2025). Performing endoscopy within 12–24 hours after ingestion and applying the Zargar classification are crucial for evaluating the condition and guiding subsequent treatment (Akkelle et al., 2023; Kara et al., 2024; Xu et al., 2024). Most studies on CEI worldwide have focused on developing countries. Detailed data on the epidemiology and clinical features of accidental corrosive ingestion in children from China’s rapidly industrializing cities are still limited. Therefore, this single-center retrospective cohort study conducted in Wuhan aimed to describe the epidemiological and clinical features of accidental corrosive ingestion in children and to identify clinical parameters associated with the severity of acute endoscopic injury.

## 2 Materials and methods

### 2.1 Study population

A retrospective analysis was conducted on the medical records of 138 pediatric patients admitted to the Department of Gastroenterology, Wuhan Children’s Hospital, for accidental ingestion of corrosive substances between July 2014 and October 2024.

Inclusion criteria were as follows (1): a clear history of accidental corrosive ingestion with hospital presentation within 48 hours of ingestion (defined as the acute phase) (2); age ≤18 years (3); completion of the first endoscopic examination within 48 hours of admission; and (4) availability of complete clinical data, including at least age, sex, time from ingestion to presentation, and endoscopic findings.

Exclusion criteria were as follows (1): a previous history of corrosive ingestion or known esophageal stricture (2); concurrent severe esophageal diseases (e.g., esophageal atresia, hiatal hernia, achalasia) or congenital gastrointestinal malformations (3); a history of upper gastrointestinal surgery (excluding nasogastric tube placement) (4); failure to undergo endoscopic examination or endoscopy performed >48 hours after admission; and (5) missing key clinical data (e.g., unclear time of ingestion, indeterminate endoscopic findings).

### 2.2 Research methods

#### 2.2.1 Data collection

The following data were extracted from the electronic medical record system (1): general patient information: age, sex, place of residence, and cause of exposure; (2) ingestion details: time interval from ingestion to medical presentation, chemical property (alkaline, acidic, or neutral) and physical form(liquid, solid, powder, etc.) of the corrosive substance; (3) pre-hospital management measures;(4) acute-phase symptoms;(5) findings from the initial endoscopic examination; (6) emergency pharmacological treatment administered; (7) long-term complications.

Due to the inherent limitations of this retrospective study, key variables including the specific concentration, ingested volume, and detailed chemical composition of the corrosive agents were not fully recorded. The absence of these variables may affect the assessment of injury severity and the analysis of related factors; therefore, the conclusions should be considered exploratory. Furthermore, the follow-up rate was notably low (87.7% lost to follow-up), and follow-up data were used only for descriptive reporting and were not included in the primary analysis.

#### 2.2.2 Endoscopic assessment and grading

The initial endoscopic examination for all enrolled children was performed at the endoscopy center of our hospital. The severity of esophageal mucosal injury was graded according to the Zargar classification criteria (Zargar et al., 1991) (**Table 1**). For statistical convenience, patients with normal mucosa on endoscopy (Grade 0) were classified into the endoscopically normal group, while those with any degree of mucosal damage (≥ Grade I) were classified into the endoscopically abnormal group. Within the abnormal group, the children were further divided into a mild group (Grades I–IIa) and a severe group (≥ Grade IIb) for exploratory subgroup analysis.

**Table 1:** Zargar grading of esophageal injury under endoscopy.

Grade	Endoscopic findings
Grade 0	Normal mucosa
Grade I	Mucosal erythema and edema
Grade IIa	Scattered superficial ulcerations, erosions, and exudate
Grade IIb	Grade IIa with isolated, circular deep ulcers (>5 mm in diameter)
Grade IIIa	Multiple deep ulcers with localized necrotic mucosa
Grade IIIb	Extensive mucosal necrosis (involving >50% of the esophageal circumference)
Grade IV	Esophageal perforation

Based on the Zargar grading criteria (Zargar et al., 1991).

#### 2.2.3 Grouping criteria

**Pre-hospital management grouping**

Group A: No pre-hospital intervention
Group B: Simple pre-hospital management (e.g., wiping the mouth with a towel or rinsing the mouth)
Group C: Active pre-hospital management (e.g., induced vomiting, ingestion of water or milk, gastric lavage at other medical institutions, or symptomatic treatment)

**Acute symptom grouping**

Group 1: Asymptomatic
Group 2: Oral symptoms only (e.g., lip swelling/pain or burning sensation in the mouth)
Group 3: Gastrointestinal symptoms (e.g., drooling, food refusal, vomiting, odynophagia, or abdominal pain)

**Chronic complication grouping**

Patients were divided based on follow-up status into a follow-up group and a lost-to-follow-up group. Based on the initial endoscopic Zargar grade, they were further stratified into a mild injury group (Grades 0–IIa) and a severe injury group (≥ Grade IIb). Given the low follow-up rate and the presence of selection bias, the results of this part were used only for descriptive analysis.

### 2.3 Statistical analysis

Data analysis was performed using R software (version 4.3.0) and Zstats. Non-normally distributed continuous data are expressed as median (interquartile range), and categorical data are presented as number (percentages). Group comparisons were conducted using the *χ*² test or Fisher’s exact test.

### 2.4 Multivariate analysis

Variables with a P < 0.05 in univariate analysis were entered into a multivariate logistic regression model, with adjustment for basic variables such as sex and place of residence, to identify independent factors associated with CEI. Results are presented as odds ratios (OR) with 95% confidence intervals (CI).

### 2.5 Subgroup analysis

1. Among children with abnormal Zargar grading on endoscopy (n=40), they were divided according to injury severity into a mild group (Grades I–IIa) and a severe group (Grades IIb–IIIb). Univariate analysis was used to compare clinical variables between the two groups to explore potential factors associated with severe injury.
2. In the follow-up analysis, due to the low follow-up rate (12.32%) and the presence of selection bias, clinical characteristics of the follow-up group and the lost-to-follow-up group were compared descriptively only, without calculating P-values. For the 17 children who completed follow-up, the incidence of complications according to different Zargar grades was also described only, without between-group statistical inference, and the complication rates were not extrapolated to the overall population.

P < 0.05 was considered statistically significant.

## 3 Results

### 3.1 General information, epidemiological and clinical characteristics

Among the 138 children with corrosive substance ingestion, the median age was 2.08 years (IQR, 1.54–3.27). Children aged ≤5 years accounted for 87.68% (121/138), while those >5 years accounted for 12.32% (17/138). The male-to-female ratio was 2:1 (92 males [66.67%] and 46 females [33.33%]). Rural children numbered 74 (53.62%) and urban children 64 (46.38%) (**Table 2**). The spatial distribution of cases showed an obvious “two-campus” clustering pattern. The top three locations were the main campus area (17 cases,15.74%), the adjacent urban-rural fringe (17 cases,15.74%), and the administrative district of the west campus (14 cases,12.96%), together accounting for 44.44% of cases. The remaining cases were scattered across other districts (see **Supplementary Figures S1**, **S2**). The median time from ingestion to hospital admission was 3.75 hours (IQR, 2–7 hours); 100 children (72.46%) sought medical care within 6 hours. The median hospital stay was 2 days (IQR, 2–4 days). Regarding pre−hospital management, 62 children (44.93%) received no intervention, 21 (15.22%) only simple measures (e.g., wiping the mouth or rinsing), and 55 (39.86%) received active pre−hospital management (including induced vomiting, drinking water/milk, or gastric lavage at another facility). Acute−phase symptoms were predominantly gastrointestinal (58 cases, 42.03%), followed by oral symptoms (41 cases, 29.71%); asymptomatic cases accounted for 39 (28.26%) (**Table 2**).

**Table 2:** Demographic and acute-phase clinical baseline characteristics of 138 children with CEI.

Clinical characteristics	Group / statistical measures	n(%)/M(Q_1_, Q_3_;)
Age (years)	M (Q_1_, Q_3_;)	2.08 (1.54–3.27)
Age group	≤5 years	121 (87.68)
	>5 years	17 (12.32)
Sex	Male	92 (66.67)
	Female	46 (33.33)
Residence	Urban	64 (46.38)
	Rural	74 (53.62)
Exposure cause	Accidental ingestion	138 (100.00)
	Self-harm	0 (0.00)
Hospital stay (days)	M (Q_1_, Q_3_;)	2.00 (2.00–4.00)
Time from ingestion to presentation (hours)	M (Q_1_, Q_3_;)	3.75 (2.00–7.00)
Time group	≤6 h	100 (72.46)
	>6 h	38 (27.54)
Pre-hospital management^a^	Group A	62 (44.93)
	Group B	21 (15.22)
	Group C	55 (39.86)
Acute-phase symptoms^b^	Group 1	39 (28.26)
	Group 2	41 (29.71)
	Group 3	58 (42.03)

Data are presented as number (percentage) unless otherwise specified; M: median, Q_1_: first quartile, Q_3_;: third quartile.^a^Group A, no intervention; Group B, simple pre−hospital measures; Group C, pre−hospital management.^b^Group 1, asymptomatic; Group 2, oral symptoms only; Group 3, gastrointestinal symptoms.

### 3.2 Characteristics of corrosive agents, endoscopic grading, and treatment

**Figure 1:**
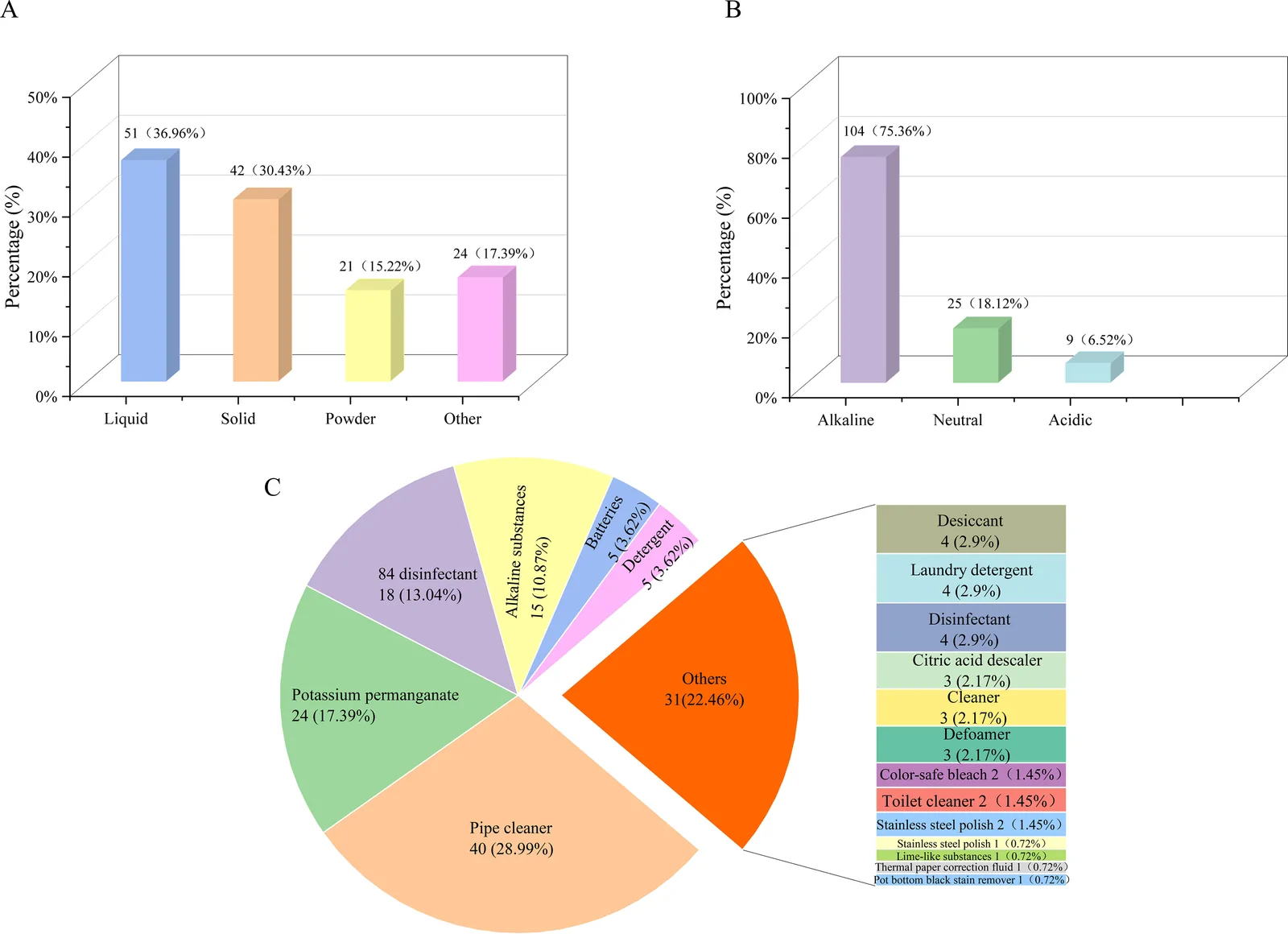
Analysis of Physicochemical characteristics of corrosive agents. **(A)** Classification of corrosive agents by physical form. **(B)** Classification of corrosive agents by chemical property. **(C)** Classification of corrosive agents by specific type.

**Figure 2:**
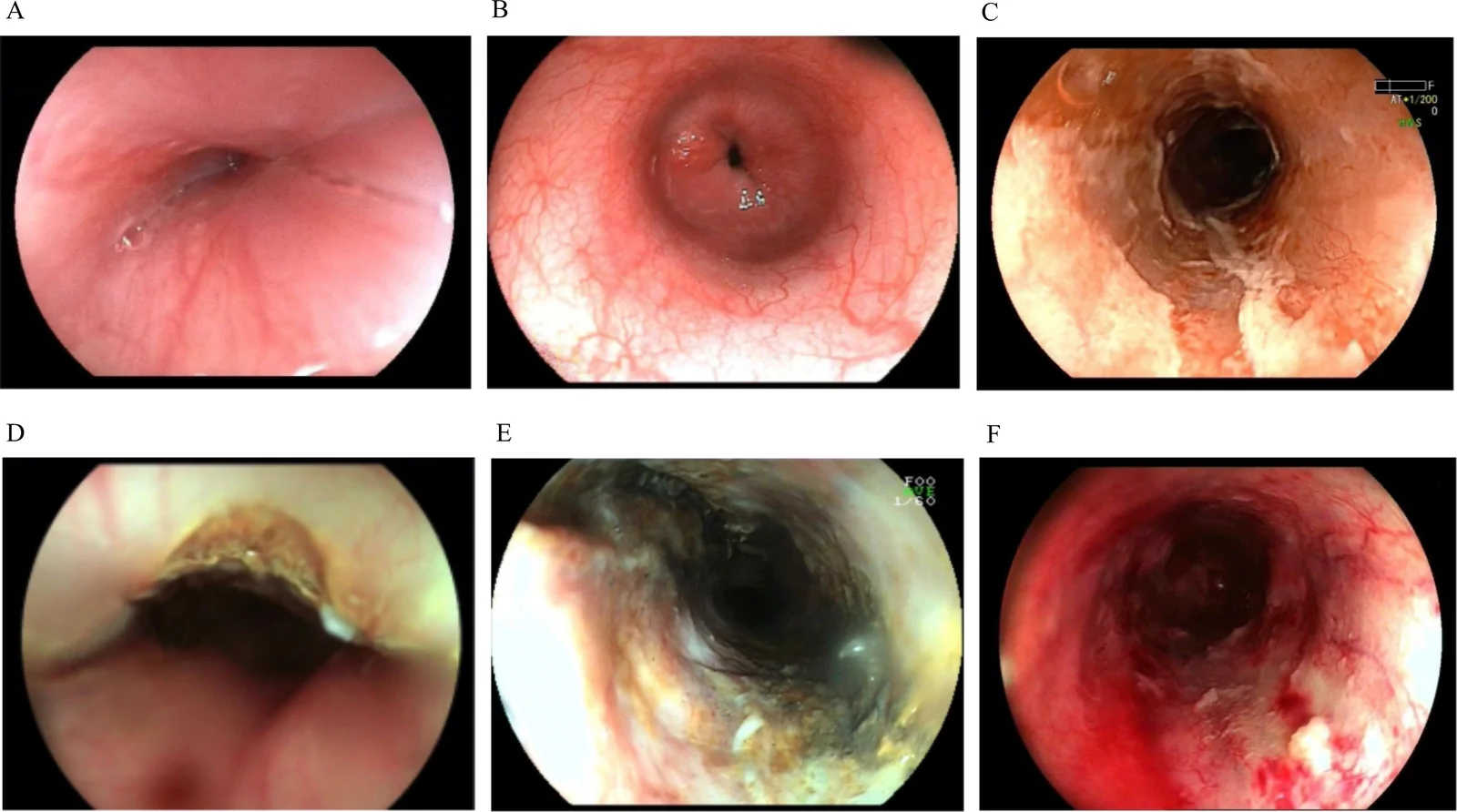
Typical Endoscopic findings of Zargar grading in children with CEI. **(A)** Grade 0, normal mucosa. **(B)** Grade I, mucosal erythema and edema. **(C)** Grade IIa, erosions with overlying white exudate in the mid-esophagus. **(D)** Grade IIb, a deep ulcer in the esophageal mucosa. **(E)** Grade IIIa, extensive erosions, ulcers, and adherent yellowish-brown necrotic material throughout the esophageal segment. **(F)** Grade IIIb, large areas of white exudate and erosions involving over half of the esophageal circumference, with an obscured vascular pattern.

Among the corrosive agents ingested by the 138 children, liquid agents accounted for 51 cases (36.96%), solid agents for 42 cases (30.43%), other formulations for 24 cases (17.39%), and powder agents for 21 cases (15.22%). Based on pH, alkaline agents were most common (104 cases, 75.36%), followed by neutral agents (25 cases, 18.12%) and acidic agents (9 cases, 6.52%). In terms of specific types, drain cleaners were the predominant agent (40 cases, 28.99%), followed by potassium permanganate (24 cases, 17.39%), sodium hypochlorite (84 disinfectant) and similar products (18 cases, 13.04%), and other alkaline substances (15 cases, 10.87%) (**Table 3**; **Figure 1**). All children underwent early endoscopic examination. The initial endoscopic Zargar grading revealed that 98 cases (71.01%) were Grade 0 (i.e., normal esophageal mucosa). Mucosal injury (Grades I - IIIb) was observed in 40 cases (28.99%), specifically: Grade I in 3 cases (2.17%), Grade IIa in 28 cases (20.29%), Grade IIb in 1 case (0.72%), Grade IIIa in 5 cases (3.62%), and Grade IIIb in 3 cases (2.17%). Representative endoscopic findings are shown in **Figure 2**. Regarding treatment, antibiotics were administered to 39 patients (28.26%), proton pump inhibitors (PPIs) to 127 patients (92.03%), and corticosteroids to 48 patients (34.78%). Among those receiving corticosteroids, budesonide was administered in 38 cases (27.54%) and prednisone in 10 cases (7.24%) (**Table 3**).

**Table 3:** Characteristics of corrosive agents, Endoscopic grading, and treatment.

Characteristic	Category	Group	n(%)
Corrosive agent	Physical form	Liquid	51 (36.96)
		Solid	42 (30.43)
		Powder	21 (15.22)
		Other^*^	24 (17.39)
	Chemical nature	Alkaline	104 (75.36)
		Neutral	25 (18.12)
		Acidic	9 (6.52)
Initial endoscopic finding	Zargar grade	Grade 0	98 (71.01)
		Grade I	3 (2.17)
		Grade IIa	28 (20.29)
		Grade IIb	1 (0.72)
		Grade IIIa	5 (3.62)
		Grade IIIb	3 (2.17)
Treatment	Antibiotics	Yes	39 (28.26)
		No	99 (71.74)
	Corticosteroids	Yes	48 (34.78)
		No	90 (65.22)
	Type of corticosteroid^a^	Budesonide	38 (27.54)
		Prednisone	10 (7.24)
	Acid suppressant (PPI)^b^	Yes	127 (92.03)
		No	11 (7.97)

Data are presented as number (percentage).*Other includes gel, paste, etc.^a^Analysis restricted to patients who received corticosteroids; categorized by the primary route of administration (local or systemic).^b^PPI, proton pump inhibitor.

### 3.3 Association analysis of factors related to corrosive ingestion and abnormal endoscopic Zargar grading

**Figure 3:**
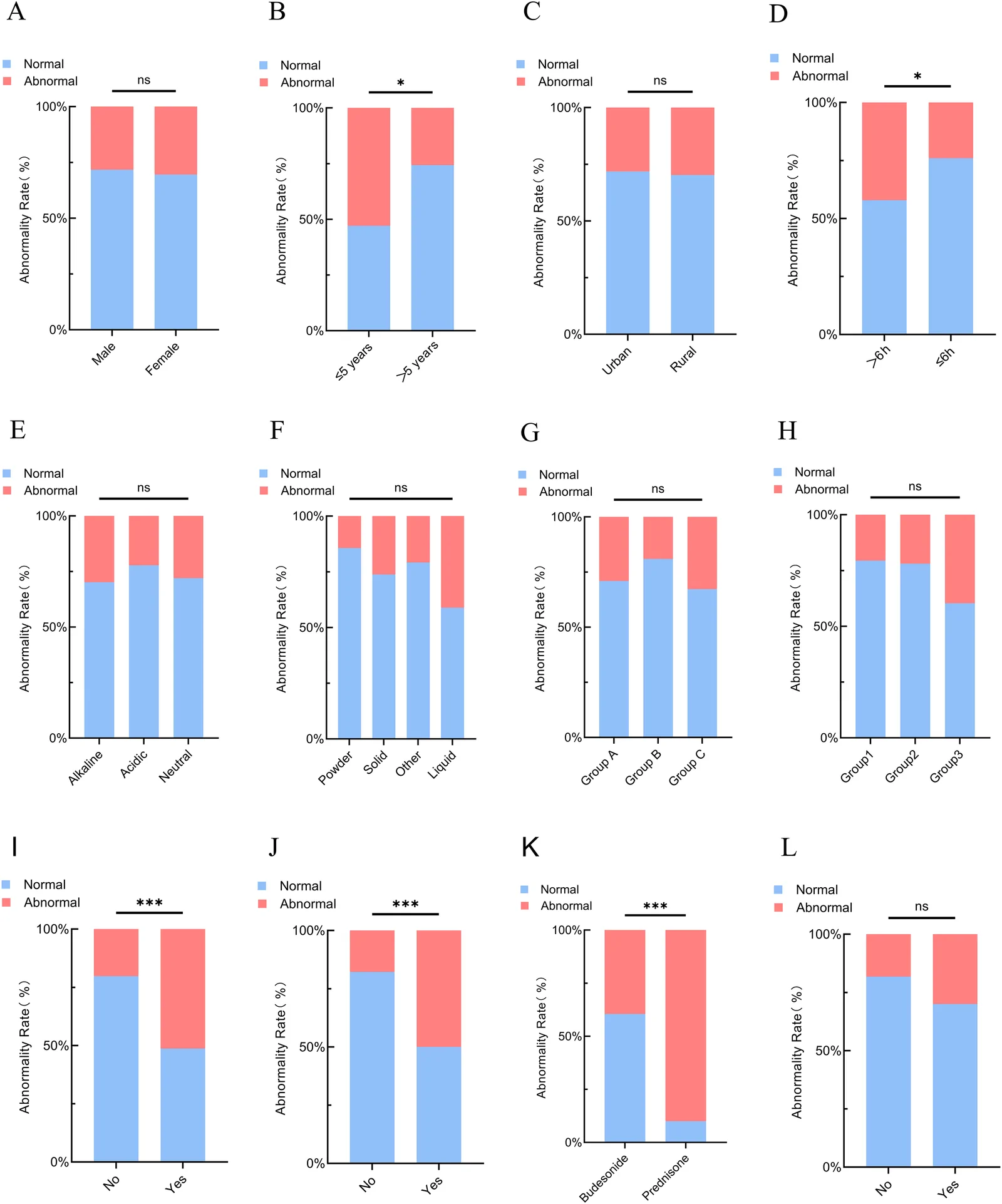
Univariate analysis of risk factors associated with abnormal Endoscopic grading in cases of injury **(A)** Sex; **(B)** Age; **(C)** Residence; **(D)** Time from ingestion to admission; **(E)** Corrosive agent property (Chemical nature); **(F)** Corrosive agent type (Physical form); **(G)** Pre−hospital management; **(H)** Acute−phase symptoms; **(I)** Antibiotic use; **(J)** Corticosteroid use; **(K)** Type of corticosteroid; **(L)** Acid suppressant use. In the figure, ns indicates no statistical significance, * denotes P < 0.05 and *** denotes P<0.001.

This study included 138 children with corrosive substance ingestion. Among them,40 cases (28.99%) were in the abnormal endoscopic Zargar grading group, and 98 cases (71.01%) were in the normal group. Univariate analysis showed that age and time from ingestion to hospital presentation were significantly associated with abnormal endoscopic Zargar grading. Children older than 5 years represented 22.50% (9/40) of the abnormal group versus 8.16% (8/98) of the normal group (*χ*² = 4.160, *P* = 0.041). Delayed presentation (>6 hours) occurred in 40.00% (16/40) of the abnormal group compared with 22.45% (22/98) of the normal group (*χ*² = 4.385,*P* = 0.036). Among corrosive agent types, liquid agents had a higher proportion of injury (52.50% vs. 7.50% for powder), but this difference did not reach statistical significance (*P* = 0.078). Sex, residence, pre-hospital management, acute symptoms, corrosive agent type, and corrosive agent property were not associated with abnormal endoscopic grading (all *P* > 0.05) (**Table 4**; **Figure 3**).

**Table 4:** Correlation analysis between risk factors for accidental ingestion of corrosive substances and abnormal endoscopic Zargar grading (n=138).

Characteristic		Endoscopic normal (n=98)	Endoscopic abnormality (n=40)	*χ*²	*P*
Age	≤5 years	90 (91.84)	31 (77.50)	4.160	0.041^*#^
	>5 years	8 (8.16)	9 (22.50)		
Sex	Male	66 (67.35)	26 (65.00)	0.070	0.791
	Female	32 (32.65)	14 (35.00)		
Residence	Urban	46 (46.94)	18 (45.00)	0.043	0.836
	Rural	52 (53.06)	22 (55.00)		
Time from ingestion to presentation	>6h	22 (22.45)	16 (40.00)	4.385	0.036^*^
	≤6h	76 (77.55)	24 (60.00)		
Pre-hospital management^a^	Group A	44 (44.90)	18 (45.00)	1.382	0.501
	Group B	17 (17.35)	4 (10.00)		
	Group C	37 (37.76)	18 (45.00)		
Acute-phase symptoms^b^	Group 1	31 (31.63)	8 (20.00)	5.553	0.062
	Group 2	32 (32.65)	9 (22.50)		
	Group 3	35 (35.71)	23 (57.50)		
Corrosive agent type (Physical form)	Powder	18 (18.37)	3 (7.50)	6.821	0.078
	Solid	31 (31.63)	11 (27.50)		
	Other	19 (19.39)	5 (12.50)		
	Liquid	30 (30.61)	21 (52.50)		
Corrosive agent property (Chemical nature)	Alkaline	73 (74.49)	31 (77.50)	0.246	0.884^#^
	Acidic	7 (7.14)	2 (5.00)		
	Neutral	18 (18.37)	7 (17.50)		
Antibiotics	No	79 (80.61)	20 (50.00)	13.130	<0.001^***^
	Yes	19 (19.39)	20 (50.00)		
Corticosteroids	No	74 (75.51)	16 (40.00)	15.790	<0.001^***^
	Yes	24 (24.49)	24 (60.00)		
Type of corticosteroid	Budesonide	23 (23.47)	15 (37.50)	25.609	<0.001^***^
	Prednisone	1 (1.02)	9 (22.50)		
Acid suppressant (PPI)^c^	No	9 (9.18)	2 (5.00)	0.227	0.633^#^
	Yes	89 (90.82)	38 (95.00)		

Data are presented as number (percentage). The χ² test was used unless otherwise specified. ^#^Fisher's exact test was used. *P<0.05,***P<0.001,indicating statistical significance. ^a^Pre-hospital management: Group A, no intervention; Group B, simple pre-hospital measures; Group C, pre-hospital management. ^b^Group 1, asymptomatic; Group 2, oral symptoms only; Group 3, gastrointestinal symptoms. ^c^PPI, proton pump inhibitor.

Univariate analysis of treatment-related variables (antibiotics, corticosteroids, and acid suppressants) showed that the usage rates of antibiotics and corticosteroids were significantly higher in the abnormal grading group than in the normal group (both *P* < 0.001), whereas there was no significant difference in the use of acid suppressants between the two groups (*P* > 0.05). It should be noted that the use of antibiotics and corticosteroids mainly reflected the clinicians’ judgment of injury severity rather than being independent factors associated with injury. Therefore, these variables were not included as predictors in the subsequent multivariate analysis.

### 3.4 Multivariate logistic regression analysis of factors associated with abnormal endoscopic findings

Variables with a *P* < 0.05 in univariate analysis were entered into a multivariate logistic regression model, with adjustment for sex and place of residence as baseline variables. The results showed that age ≤ 5 years was an independent protective factor against abnormal endoscopic grading (OR = 0.30; 95% CI: 0.10–0.86; *P* = 0.025). Time from ingestion to hospital presentation ≤ 6 hours was also an independent protective factor (OR = 0.43; 95% CI: 0.19–0.96; *P* = 0.038) (**Table 5**). Conversely, age > 5 years and delayed presentation >6 hours were positively associated with an increased risk of CEI (OR = 3.33 and 2.33, respectively).

**Table 5:** Multivariate logistic regression analysis of factors associated with Esophageal injury following accidental corrosive ingestion in children(n=138).

Variable	β	S.E	Z	OR (95%CI)	P
Constant	0.73	0.58	1.26	2.07 (0.67-6.46)	0.208
Age (≤5 years vs. >5 years)	-1.21	0.54	-2.24	0.30 (0.10-0.86)	0.025^*^
Time from ingestion to presentation (≤6 h vs. >6 h)	-0.85	0.41	-2.07	0.43 (0.19-0.96)	0.038^*^

OR, odds ratio; CI, confidence interval; SE, standard error. Reference groups: age >5 years and time from ingestion to hospital presentation >6 hours. An OR <1 indicates a protective effect (i.e., age ≤5 years and time to presentation ≤6 hours were associated with a lower risk of abnormal endoscopic findings). Conversely, age >5 years and delayed presentation >6 hours were associated with an increased risk (OR = 3.33 and 2.33, respectively).*P <0.05 was considered statistically significant.

### 3.5 Exploratory analysis of mild and severe esophageal injury within the abnormal group

To further explore factors associated with the severity of esophageal injury, the 40 children with abnormal endoscopic grading were analyzed. Among them, 31 cases(77.50%) were in the mild injury group (Grade I–IIa),and 9 cases (22.50%) were in the severe injury group (≥ Grade IIb).Univariate analysis showed that none of the clinical characteristics(age, sex, place of residence, time to presentation, pre-hospital management, acute symptoms, type of corrosive agent, or chemical property of the corrosive agent) were significantly different between the two groups (all *P* > 0.05) (**Table 6**).

**Table 6:** Univariate analysis of factors associated with Esophageal injury severity in children with abnormal Endoscopic findings (n=40).

Characteristic		Mild (n=31)	Severe (n=9)	*χ*²	*P*
Age	>5 years	8 (25.81)	1 (11.11)	0.227	0.634
	≤5 years	23 (74.19)	8 (88.89)		
Sex	Male	21 (67.74)	5 (55.56)	0.077	0.781
	Female	10 (32.26)	4 (44.44)		
Residence	Urban	13 (41.94)	5 (55.56)	0.117	0.732
	Rural	18 (58.06)	4 (44.44)		
Time from ingestion to presentation	>6h	13 (41.94)	3 (33.33)	0.006	0.938
	≤6h	18 (58.06)	6 (66.67)		
Pre-hospital management^a^	Group A	15 (48.39)	3 (33.33)	-	0.467^#^
	Group B	4 (12.90)	0 (0.00)		
	Group C	12 (38.71)	6 (66.67)		
Acute-phase symptoms^b^	Group 1	5 (16.13)	3 (33.33)	-	0.388^#^
	Group 2	8 (25.81)	1 (11.11)		
	Group 3	18 (58.06)	5 (55.56)		
Corrosive agent type (Physical form)	Powder	3 (9.68)	0 (0.00)	-	0.582^#^
	Solid	8 (25.81)	3 (33.33)		
	Other	5 (16.13)	0 (0.00)		
	Liquid	15 (48.39)	6 (66.67)		
Corrosive agent property (Chemical nature)	Alkaline	23 (74.19)	8 (88.89)	-	1.000^#^
	Acidic	2 (6.45)	0 (0.00)		
	Neutral	6 (19.35)	1 (11.11)		

^a^;Pre-hospital management groups (A: no intervention; B: simple measures; C: active management). ^b^Acute-phase symptom groups (1: asymptomatic; 2: oral symptoms only; 3: gastrointestinal symptoms). ^#^Fisher's exact test. P < 0.05 was considered statistically significant.

### 3.6 Descriptive analysis of chronic complications

Among the 138 children, only 17 (12.32%) returned for follow-up within 2–4 weeks after ingestion. A comparison of clinical characteristics between the follow-up group and the lost-to-follow-up group is shown in **Table 7** (descriptive analysis only; no statistical tests were performed). In the follow-up group, the median hospital stay was 8.0 days, whereas in the lost-to-follow-up group it was 2.0 days. Severe esophageal injury (≥ Grade IIb) accounted for 66.67% in the follow-up group, compared with 33.33% in the lost-to-follow-up group. These differences suggest the presence of a significant selection bias: children with more severe conditions were more likely to return for follow-up. Therefore, the complication rates observed in the follow-up group should not be generalized to the entire study population.

**Table 7:** Clinical characteristics of the follow-up and lost-to-follow-up groups.

Characteristic		Total (n=138)	Lost-to-follow-up (n=121)	Follow-up (n=17)
Age (years)	M(Q_1_, Q_3_;)	2.08 (1.54, 3.27)	2.07 (1.54, 3.39)	2.20 (1.54, 2.86)
Length of hospital stay (days)	M (Q_1_, Q_3_;)	2.00 (2.00, 4.00)	2.00 (1.00, 3.00)	8.00 (4.00, 12.00)
Time from ingestion to presentation (h)	M (Q_1_, Q_3_;)	3.75 (2.00, 7.00)	3.50 (2.00, 7.00)	4.00 (2.00, 8.00)
Sex	Male	92 (66.67)	82 (67.77)	10 (58.82)
	Female	46 (33.33)	39 (32.23)	7 (41.18)
Residence	Urban	64 (46.38)	56 (46.28)	8 (47.06)
	Rural	74 (53.62)	65 (53.72)	9 (52.94)
Corrosive agent property (Chemical nature)	Alkaline	104 (75.36)	90 (74.38)	14 (82.35)
	Acidic	9 (6.52)	8 (6.61)	1 (5.88)
	Neutral	25 (18.12)	23 (19.01)	2 (11.76)
Zargar endoscopic grade	Mild^*^	129 (93.48)	118 (97.52)	11 (64.70)
	Severe^#^	9 (6.52)	3 (2.48)	6 (35.30)

Data are presented as number (percentage) unless otherwise specified. M: median; Q_1_: first quartile; Q_3_;: third quartile. ^*^Mild injury: Grade 0–IIa; ^#^Severe injury: ≥ Grade IIb. No statistical comparisons were performed between groups. This table is for descriptive purposes only.

Among the 17 children in the follow-up group, 9 (52.94%) developed long-term dysphagia and 8 (47.06%) developed esophageal strictures. According to the initial endoscopic Zargar grading, there were 11 children in the mild injury group and 6 in the severe injury group. The complication rates for each group are shown in **Table 8**. In the mild injury group, 3 cases (27.27%) developed dysphagia and 2 cases (18.18%) developed esophageal stricture. In the severe group, all 6 cases (100%) developed both dysphagia and esophageal stricture. Due to the small sample size (n = 17) and the presence of selection bias, the above results are reported descriptively only, and no between-group statistical inference was made.

**Table 8:** Short-term complication rates according to Zargar grade in 17 followed-up children.

Short-term complication		Mild group(n = 11)^*^	Severe group(n = 6)^#^
Dysphagia	No	8 (72.73)	0 (0.00)
	Yes	3 (27.27)	6 (100.00)
Esophageal Stricture	No	9 (81.82)	0 (0.00)
	Yes	2 (18.18)	6 (100.00)

Data are presented as number (column percentage). Due to the low follow-up rate (12.3%) and selection bias, no statistical comparisons were performed. Results are for descriptive purposes only and cannot be generalized.^*^Mild injury: Grade 0–IIa; ^#^Severe injury: ≥ Grade IIb.

## 4 Discussion

Ingestion of corrosive substances not only causes acute esophageal injury but can also lead to late-stage esophageal stricture; furthermore, the long-term risk of esophageal cancer increases by more than 1000-fold, posing a serious threat to children’s health (Eskander et al., 2019). This study has two core findings. First, age >5 years and time from ingestion to hospital presentation >6 hours were associated with an increased risk of acute esophageal injury after accidental corrosive ingestion (adjusted OR = 3.33 and 2.33, respectively). Second, 28.26% of the children were asymptomatic in the acute phase, although endoscopic examination nevertheless revealed mucosal injury. These results suggest that for children with suspected corrosive ingestion, endoscopic evaluation should be performed promptly, even in the absence of typical symptoms.

### 4.1 Epidemiological features and local characteristics

In our cohort, most children were under 5 years of age (87.68%), the male−to−female ratio was 2:1, and alkaline agents accounted for 75.36%, with drain cleaners being the most common (28.99%). These figures align with global trends (Arnold and Numanoglu, 2017; Chirica et al., 2017; Almajed et al., 2024). The high proportion of children under 5 years reflects their strong curiosity, poor risk recognition, and lack of danger awareness at this age. Common household corrosive products are often drain cleaners and other strong alkaline cleaning liquids (Jiang and Gong, 2023). Their main components, such as sodium hydroxide, are strong alkalis that can cause liquefactive necrosis and even perforation (Kalayarasan et al., 2019; Li et al., 2022). The cases were clustered around the two hospital campuses and the adjacent urban−rural fringe, which may indicate inadequate supervision and improper storage of corrosive agents in these areas. In the context of rapid urbanization, extending health education and medical resources to these high−risk areas, along with strengthening family education, may help improve outcomes.

### 4.2 Mechanisms underlying the increased risk associated with age and time to presentation

Age > 5 years was associated with a higher risk of esophageal injury. Older children have a larger oral cavity and a more coordinated swallowing reflex, allowing them to swallow larger amounts of corrosive agent more quickly and making them less likely to expel it. In contrast, children under 5 years of age have smaller mouths and often cry vigorously and refuse to swallow because of oropharyngeal pain after ingestion, which may lead to expulsion of part of the corrosive agent and thus reduce the amount that reaches the esophagus (Huang et al., 2024). However, once injury occurs in younger children, the risk of long−term stricture is higher (Radhakrishna et al., 2022).

Presentation > 6 hours after ingestion was another factor associated with increased risk, consistent with the findings of Rafeey et al. (2016). Pathophysiologically, corrosive agents cause tissue damage within seconds of contact with the esophageal mucosa; within the first 24 hours, hemorrhage, edema, and even necrosis may occur. Esophageal repair begins around days 10–15 after injury, and scar contracture may continue for months (Chirica et al., 2017). Therefore, seeking medical attention within 6 hours provides a critical window for early endoscopic intervention (Chinese Medical Association et al., 2025; Kim et al., 2025).

### 4.3 Occult injury in asymptomatic children and endoscopic grading characteristics

This study demonstrated that acute-phase clinical manifestations were not entirely consistent with endoscopic Zargar grading (Fernandez et al., 2020). Although gastrointestinal symptoms were the most common presentation (42.03%), some children who were asymptomatic or presented with only oral symptoms nevertheless had esophageal mucosal injury. Univariate analysis showed only a borderline association between acute symptoms and abnormal endoscopic findings (*P* = 0.062), suggesting that relying solely on clinical symptoms may underestimate the actual severity (Ngobese et al., 2022; Kouhnavard et al., 2023). This occult nature of CEI supports the use of routine endoscopy, rather than delaying or canceling the procedure based on the absence of symptoms.

Most children (71.01%) had normal mucosa on endoscopy, possibly attributable to the strong irritant effect on the oropharyngeal mucosa and the small volumes of corrosive agent ingested by young children. Among those with mucosal abnormalities, Grade IIa injury was the most common (20.29%), which may be related to the saponifying effect of alkaline agents on adipose tissue.

### 4.4 Effect of the physical form of the corrosive agent

Liquid agents accounted for a higher proportion of the abnormal endoscopic group than powders or solids (52.50% vs. 7.50% and 26.19%, respectively), although the difference did not reach statistical significance (*P* = 0.078). Liquid corrosive agents are fluid and permeable; they pass easily through the esophagus and may transiently accumulate at physiological narrowing, prolonging contact time and thus exacerbating injury (Khan et al., 2023; Abou et al., 2024). Because of the limited sample size and missing data on concentration and dose, this finding needs confirmation in larger studies.

### 4.5 Subgroup analysis of mild versus severe injury

To further explore factors associated with the severity of esophageal injury, we divided the 40 children with abnormal endoscopic findings into a mild injury group (Grades I–IIa, n = 31) and a severe injury group (≥ Grade IIb, n = 9). Univariate analysis showed no significant differences between the two groups in age, time to presentation, type of corrosive agent, or other clinical variables (all P > 0.05). This suggests that age and delayed presentation mainly affect the occurrence of injury, rather than its severity. Once the mucosa is already damaged, these factors have limited value in distinguishing mild from severe injury. The literature rarely uses age as an independent predictor of injury severity; most studies emphasize that pH, concentration, ingested dose, and contact time are the core determinants (Irlayıcı et al., 2025). Moreover, the severe group contained only nine cases, providing insufficient statistical power, and the lack of key exposure data (e.g., concentration and ingested dose) precludes definitive conclusions.

### 4.6 Treatment patterns

The rates of corticosteroid and antibiotic use were significantly higher in the abnormal endoscopic group than in the normal group, a finding that mainly reflects clinical decisions based on injury severity. The use of corticosteroids to prevent esophageal stricture remains controversial (Iizuka et al., 2018; Key, 2022; Zou et al., 2022). In contrast, proton pump inhibitors (PPIs) are used to reduce secondary injury from acid reflux; the use rate in this cohort was 92.03%, consistent with current recommendations (Niedzielski et al., 2020; Chinese Medical Association et al., 2025).

### 4.7 Follow−up bias and its implications

The follow−up rate in this study was notably low (12.32%). Compared with the lost−to−follow−up group, children who returned for follow−up had longer hospital stays and a higher proportion of severe injury (66.67% vs. 33.33%), indicating the presence of severe selection bias. Therefore, the rates of esophageal stricture (47.06%) and dysphagia (52.94%) reported here are likely overestimated and should not be generalized to the entire study population.

Notably, two cases of esophageal stricture were observed during follow−up in the mild injury group (Grades 0–IIa), suggesting that a normal or mildly abnormal initial endoscopy does not guarantee the absence of late stricture. Another child in the mild injury group presented with dysphagia at follow−up, but repeat endoscopy and an iodine contrast study showed no evidence of stricture; this might be explained by decreased esophageal compliance or impaired peristalsis, and continued follow−up is warranted. These observations underscore the need for regular follow−up of all children with a history of corrosive ingestion.

### 4.8 Study limitations

This single−center retrospective study has inherent limited generalizability. Key exposure parameters (concentration, ingested volume, and chemical composition of the corrosive agent) were missing, which may introduce confounding. For example, a high−concentration alkaline agent (e.g., sodium hydroxide >10%) can cause transmural necrosis within seconds, whereas a low−concentration solution may cause only superficial erosion (Irlayıcı et al., 2025). The ingested volume directly affects the contact area and duration; even a solid or powdered agent can dissolve and form a high−concentration solution when swallowed in large amounts, thereby worsening the injury (Rogalidou, 2026). Different chemical components (sodium hydroxide, sodium hypochlorite, oxalic acid, etc.) have different tissue affinities and mechanisms of injury, but in this study, we combined them into broad “alkaline” or “acidic” categories, which may not capture their true effects (Irlayıcı et al., 2025). These missing data may lead to residual confounding, effect modification, and selective reporting bias.

In addition, the low follow−up rate and the baseline differences between the follow−up and lost−to−follow−up groups imply that the complication data should not be considered representative of the overall population. Future multicenter prospective studies with systematic collection of physicochemical parameters and complete follow−up are required.

## 5 Conclusion

In summary, following accidental corrosive ingestion in children, age > 5 years and time to presentation > 6 hours are associated with an increased risk of acute esophageal injury: the risk for children older than 5 years is approximately three−fold higher than that for children aged ≤ 5 years, and the risk for those presenting after 6 hours is approximately two−fold higher than that for those presenting within 6 hours. Moreover, 28.26% of children who were asymptomatic in the acute phase had endoscopic evidence of esophageal mucosal injury, indicating that clinical symptoms alone may not reliably predict esophageal damage. These findings are limited by the retrospective design and therefore await confirmation in prospective studies.

## Data Availability

The original contributions presented in the study are included in the article/**Supplementary Material**. Further inquiries can be directed to the corresponding author.
