# Supplementary material for: Epidemiological and clinical characteristics of pediatric corrosive ingestion and factors associated with acute esophageal injury: a single-center retrospective study
Source: Front Physiol. 2026 May 29;17:1807937. doi: 10.3389/fphys.2026.1807937 (PMC13261175; doi:10.3389/fphys.2026.1807937)
Supplement: Supplementary file 1 [file DataSheet1.pdf]

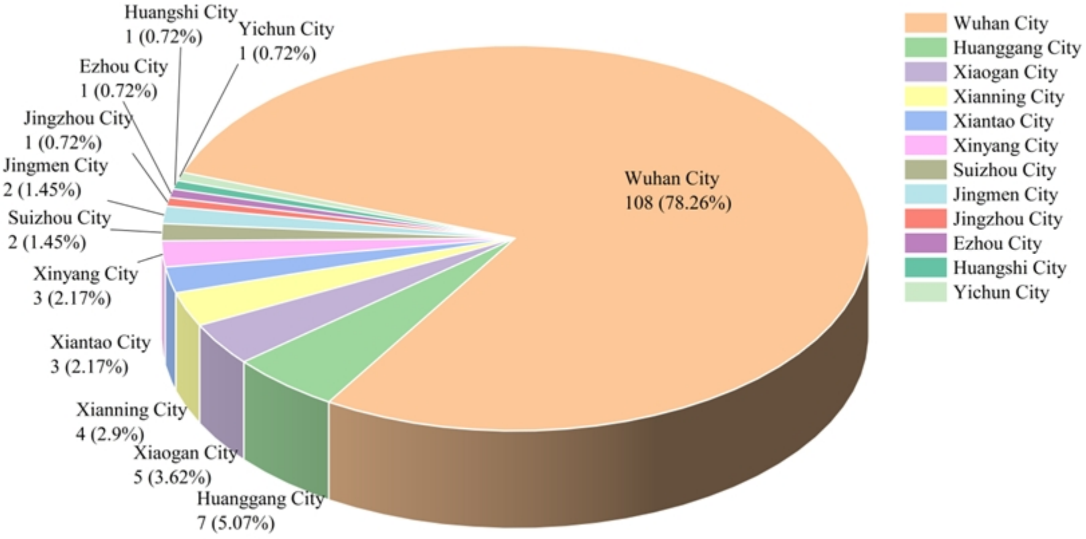

Figure S1. Regional Distribution of 138 Pediatric Cases of Accidental Corrosive Ingestion in Hubei Province

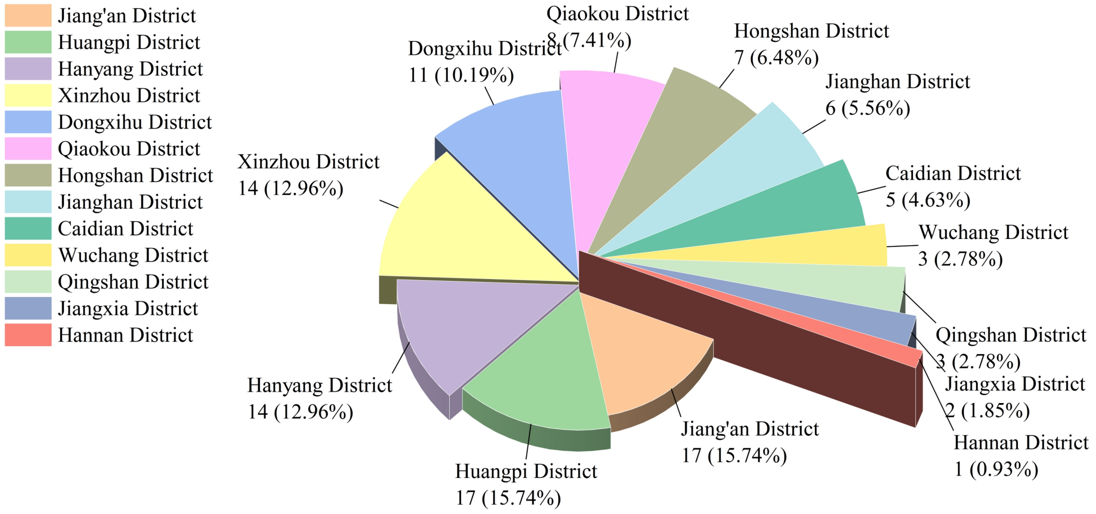

Figure S2. Distribution of Pediatric Cases of Accidental Corrosive Ingestion in Wuhan City
